# Evoregions of fleas and their small mammalian hosts: Do they coincide?

**DOI:** 10.1017/S0031182023000884

**Published:** 2023-09

**Authors:** Boris R. Krasnov, Georgy I. Shenbrot

**Affiliations:** Mitrani Department of Desert Ecology, Swiss Institute for Dryland Environmental and Energy Research, Jacob Blaustein Institutes for Desert Research, Ben-Gurion University of the Negev, Midreshet Ben-Gurion, Israel

**Keywords:** evoregions, fleas, hosts, phylogeny, spatial distribution

## Abstract

Combining the biogeography and phylogenetic patterns of parasite-host associations allows a better understanding of the history of parasite–host interactions, which can be achieved *via* biogeographic regionalization incorporating phylogenetic information. Recently, the concepts of evoregions (regions where a majority of species evolved from one or several ancestors inhabiting these regions) and evolutionary transition zones (regions of high phylogenetic turnover) have been proposed, coupled with a classification approach for these concepts. We applied this approach to 206 flea species and 265 host species of the Palearctic and aimed to identify evoregions and evolutionary transition zones for both fleas and hosts and to understand whether these evoregions and transition zones match each other. We identified 5 evoregions with 3 transition zones for either fleas or hosts, but neither the positions and boundaries of the flea and host evoregions nor the transition zones coincided. Indications of multiple geographic centres of diversification of the same flea lineages suggested that (a) the common evolutionary history of fleas and hosts was characterized by multiple events other than codiversification and that (b) dispersal played an important role in flea and host assemblies. Barriers to dispersal could be represented by landscape features (deserts and mountain ranges) and/or climate differences.

## Graphical abstract

**Figure:**
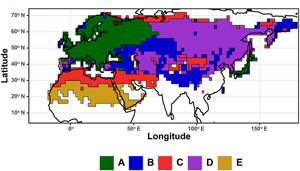

## Introduction

It is commonly known that parasite evolution is tightly linked to the evolution of their hosts. More than 100 years ago, Heinrich Fahrenholz (1913) stated that the phylogenies of parasites and their hosts are expected to be congruent if speciation in parasite lineages is a response to speciation in the lineages of their hosts (= cospeciation = codiversification; see glossaries in the study by Althoff et al., 2014; Blasco-Costa et al., 2021). This idea was further formulated as Fahrenholz's Rule (Dogiel, 1941; Stammer, 1957; Brooks, 1979; Poulin, 2007). However, the lack of appropriate analytical methods did not allow for explicitly testing various hypotheses of parasite–host coevolution until the late 1980s. Then, numerous studies of cophylogeny (i.e. the study of concordance between the phylogenies of 2 groups of interacting species; Blasco-Costa et al., 2021) in many host–parasite systems were carried out (e.g. Hafner and Nadler, 1988, 1990; Paterson et al., 1993; Hafner and Page, 1995; Beveridge and Chilton, 2001; Desdevises et al., 2002; Krasnov and Shenbrot, 2002). The majority of these studies have demonstrated a general lack of full congruence of parasite and host phylogenies, thus suggesting that the history of parasites and their hosts is complicated by the predominance of various evolutionary events other than codiversification.

Fleas (Insecta: Siphonaptera) are a convenient model taxon for cophylogenetic studies. This is because (a) all members of this relatively small (about 2500 species) order are obligate haematophages, (b) they are monophyletic (Whiting et al., 2008; Zhu et al., 2015), and (c) the majority of their hosts belong to a few orders of a single vertebrate class (Mammalia) (only about 6% of species parasitize birds; Medvedev and Krasnov, 2006). Although earlier narrative descriptions suggested a tight coevolution between fleas and their hosts (Traub, 1972, 1980, 1985), cophylogenetic studies provided contradictory results. Krasnov and Shenbrot (2002) demonstrated that cospeciation was generally absent in the evolutionary history of associations between fleas and their dipodid (jerboas) hosts, whereas frequent host switching and linear sorting (extinction of a parasite from a host lineage after a cospeciation event) events were widespread (see also Lu and Wu, 2005 for leptopsyllid fleas and ochotonid hosts). However, mapping of host associations on flea phylogenetic trees showed predominant associations of certain flea phylogenetic lineages with certain host phylogenetic lineages (Whiting et al., 2008). Moreover, at the large taxonomic scale, when a variety of flea and host species were considered in the analyses, the phylogenetic interaction effect appeared to be substantial and indicated that related flea species were more likely to be found on related host species (Hadfield et al., 2014; Krasnov et al., 2016). This was the case despite the fact that the degree of phylogenetic incongruence in hosts was concentrated in some, but not other, phylogenetic clades, whereas it was characteristic for the entire phylogenetic tree of fleas (Llaberia-Robledillo et al., 2023). These contradictions could arise due to the substantial influence of ecological and geographic factors on flea-host common history (Krasnov and Shenbrot, 2002), with the effect of these factors varying between biogeographic realms (Krasnov et al., 2022*a*). The results of the above-cited studies also suggest that phylogenetic congruence might arise from the matching of the deep, rather than the shallow, nodes of the interactors' phylogenetic trees (Blasco-Costa et al., 2021). Furthermore, congruent phylogenies could result from processes other than strict codiversification (Althoff et al., 2014; Russo et al., 2018). For example, phylogenetic tracking is a pattern in which speciation events in one lineage (parasites) follow speciation events in another lineage (hosts), given that the former strongly depend on the latter but not vice versa, whereas vicariance results in congruent phylogenies if both interactors are subject to similar abiotic isolation events within the same geographic region so that the isolation of populations in both interactors causes parallel branching of their trees. Blasco-Costa et al. (2021) argued that all these processes, including codiversification, are not mutually exclusive, and their combinations may produce patterns observed in nature.

The phylogenetic patterns of species associations have recently started to become an additional tool for understanding species assembly in communities of interacting partners (Corro et al., 2021; Krasnov et al., 2022*a*; Blasco-Costa et al., 2021 for review). In addition, combining biogeography and cophylogeny will allow a better understanding of the common history of these interactions because the geographical location of present species and ancestral nodes in their phylogenies might indicate hotspots of various coevolutionary events (Berry et al., 2018; Blasco-Costa et al., 2021). Another way to infer the relationship between geography and cophylogeny is to detect geographic regions accounting for the independent diversifications of either parasite or host lineages and to test for the similarity of these regions' geographic positions for parasites and their hosts. In other words, this approach involves biogeographic regionalization incorporating phylogenetic information (Holt et al., 2013; Falaschi et al., 2023). Recently, Maestri and Duarte (2020) proposed the concept of evoregions, namely biogeographic regions where the majority of species evolved from one or a few ancestors inhabiting these regions. They also introduced the concept of evolutionary transition zones as regions characterized by high phylogenetic turnover. Furthermore, Maestri and Duarte (2020) proposed an approach for classifying evoregions and evolutionary transition zones based on fuzzy logic. An advantage of using a fuzzy approach is that it allows representing the boundaries between regions as gradients (Olivero et al., 2013).

Although fleas have been thought to originate in Gondwana (Zhu et al., 2015), their diversification outburst likely happened in the Palearctic (Medvedev, 1996, 2005), resulting in the Palearctic having the highest flea species richness (about 900) as compared to other biogeographic realms (Krasnov, 2008). This high diversity could stem from 2 not mutually exclusive processes, namely *in situ* diversification (Morrone and Gutiérrez, 2005) and dispersal from other realms (Krasnov and Shenbrot, 2002; Gibert et al., 2021). Here, we applied the concept of evoregions and evolutionary transition zones to 206 species of Palearctic fleas and 265 species of their hosts for identification of evoregions and evolutionary transition zones for parasites (fleas) and their hosts (small mammals). We aimed to understand whether these evoregions and transition zones for flea and hosts match each other. Strong concordance between flea and host evoregions would indicate an important role played by cophylogeny in the common flea-host histories, whereas weak (if present at all) concordance would suggest that the predominance of host-switchings either occurred locally or followed dispersal or both.

## Materials and methods

### Geographic distributions

We selected 206 flea species for which data on geographic ranges were available (Krasnov et al., 2005, 2018; Shenbrot et al., 2007; Maestri et al., 2017). Then, we selected 266 host species (a) on which at least one of these fleas was recorded (information taken from various literature sources; see references in Krasnov et al., 2022*b*) and (b) with geographic ranges mainly situated in the Palearctic. The lists of flea and host species considered in this study are presented in Appendices 1 and 2, Supplementary Materials.

Geographic ranges for all species with 5 or more occurrence records were used as polygons. For all flea species and for rodents of the superfamily Dipodoidea, family Cricetidae, subfamily Gerbillinae, and several murine species (*Apodemus agrarius*, *A. flavicollis*, *A. mystacinus*, *A. ponticus*, *Nesokia indica*, and *Rattus pyctoris*), polygons of geographic ranges were obtained from species distribution modelling (SDM), whereas polygons of the geographic ranges of other small mammal hosts were obtained from the International Union of Nature Conservation (IUCN, 2022), corrected following Burgin et al. (2020). Further details on the calculation of geographic distributions are presented in Appendix 3, Supplementary Material.

### Phylogenies

For fleas, we used the most comprehensive molecular phylogenetic tree (Zhu et al., 2015) as a backbone. This tree contained data on all families, subfamilies, and genera (but not all species) of fleas used in this study. The positions of the species absent from the Zhu et al. (2015) tree were derived from their taxonomic positions based on morphological traits and dichotomous identification keys (Hadfield et al., 2014; see references in Krasnov et al., 2022*a*). We assigned all branch lengths to an equal length of 1 because no information on branch lengths was available; we then ultrametrized the tree using the function ‘force.ultrametric’ (with option method = ‘extend’) of the package ‘phytools’ (Revell, 2012) implemented in the R statistical environment (R Core Team, 2023).

Host phylogeny was taken as a subset of 1000 random trees from the 10 000 species-level birth-death tip-dated completed trees for 5911 mammal species of Upham et al. (2019). Then, we constructed a consensus tree using the function ‘consensus.edge’. Upham et al.'s (2019) tree for mammals is, to a certain degree, outdated, and many recently distinguished species (some dipodids, gerbillines, and arvicolines) are absent from this tree. We manually added these species to the resultant tree, and their topological positions were taken from various sources (see details and list of sources in Appendix 4, Supplementary Material). We assigned branch lengths for these species to an equal length of 1. Then, we ultrametrized the tree as described previously and resolved polytomies using the function ‘fix.poly’ implemented in the R package ‘RRphylo’ (Castiglione et al., 2018) and polytomous clades to non-zero length branches.

### Data analyses

We calculated the presence/absence of each flea or host species over a grid map of the Palearctic with 2804 cells of 2° × 2° and assigned a species' presence in each cell if the species' range covered at least 12.5% of the cell (otherwise, too many cells were excluded from the analyses, especially for species with narrow geographic distributions). The empty cells were excluded from the analyses.

Identification of evoregions for either fleas or hosts is based on the phylogenetic turnover among grid cells, measured using the phylogenetic fuzzy-weighting method (Pillar and Duarte, 2010) that takes into account both between-species phylogenetic distances and tree imbalance [i.e. the difference between the numbers of tips descending from internal nodes (Duarte et al., 2016)]. The procedure involves several steps (see details in the study by Maestri and Duarte, 2020; Nakamura et al., 2023). In brief, the first step is constructing 2 matrices, namely a matrix of pairwise phylogenetic covariances between flea or host species standardized by marginal totals (matrix *Q*; see Maestri and Duarte, 2020 for details) and a presence/absence grid cell × species matrix. Multiplication of these matrices produces a matrix of the phylogenetic composition of species assemblages in the grid cells (matrix *P*; Maestri and Duarte, 2020) that reflects phylogenetic turnover as the difference in phylogenetic composition between cells. In other words, matrix *Q* illustrates the degrees of the phylogenetic membership of each species to every other species, whereas matrix *P* is a phylogenetic fuzzy matrix reflecting the phylogenetically weighted degree of each species' belonging (ranging from 0 to 1) to each cell assemblage. Then, matrix *P* is used as an input for computing the Principal Coordinates of Phylogenetic Structure (PCPS of Duarte, 2011), which represents the Principal Coordinate Analysis (PCoA) of this matrix, using square-rooted Bray–Curtis dissimilarities between cells to avoid negative eigenvalues (Borcard et al., 2018). Gradients of phylogenetic turnover across cells are reflected in the eigenvectors produced by PCPS (Duarte et al., 2016). Subsequently, PCPS eigenvectors (using the principal components with more than 5% of explained variance) are taken as input data for the Discriminant Analysis of Principal Components based on k-means non-hierarchical clustering (DAPC; Jombart et al., 2010) that is used to perform biogeographic regionalization. The above analyses are implemented in the function ‘calc_evoregion’ of the R package ‘Herodotools’ (Nakamura et al., 2023). The optimal number of clusters obtained from DAPC is automatically calculated by the ‘calc_evoregion’ function based on the ‘elbow’ method from the R package ‘phyloregion’ (Daru et al., 2020).

After identifying evoregions, we calculated the degree of affiliation of each species assemblage in the grid cell with an evoregion that this assemblage was classified. The degree of affiliation (= degree of membership) with an evoregion varies between the assemblages of the grid cells within the evoregion because of varying phylogenetic similarity between these assemblages. This degree for a given cell was calculated as the mean phylogenetic dissimilarity between this cell and all other cells belonging to an evoregion, using the function ‘calc_affiliation_evoreg’ of the ‘Herodotools’ package. High affiliation values indicate assemblages that are highly similar to other assemblages of the same evoregion (i.e. assemblages characterized by low phylogenetic turnover), whereas low affiliation values indicate assemblages dissimilar from other assemblages of the same evoregion (i.e. assemblages characterized by high phylogenetic turnover). In other words, the latter assemblages constitute evolutionary transition zones (Maestri and Duarte, 2020).

To understand whether a particular phylogenetic lineage mainly occurs in a particular evoregion, we calculated species affiliations with evoregions using the function ‘calc_spp_association_evoreg’ of ‘Herodotools’. Because many species occurred in more than one region, we applied the approach of Maestri and Duarte (2020) (albeit less conservatively) and considered a species to belong to an evoregion if 60% of the grid cells in which this species was recorded (70% in Maestri and Duarte, 2020) were classified to this evoregion. Species that could not be affiliated with a single evoregion were denoted as widespread species (Maestri and Duarte, 2020). Then, we estimated ancestral evoregions simulating stochastic character (evoregion-specific affiliation or being widespread) maps on a phylogenetic tree of either fleas or hosts using the R package ‘phytools’.

The congruence between evoregions identified for fleas and evoregions identified for their hosts was tested for the overall similarity of their spatial structure. This was done using the *V*-measure method developed by Nowosad and Stepinski (2018) to calculate the spatial association between regionalizations and derived from a measure used in computer science for comparing different clusterings of the same domain. The *V*-measure results from comparing 2 regionalization maps and ranges from 0 to 1 (from no congruence whatsoever to perfect congruence). This measure represents a harmonic mean of 2 metrics, namely homogeneity and completeness, that also range from 0 to 1 (see details in the study by Nowosad and Stepinski, 2018). Homogeneity is the average homogeneity of the host evoregions with respect to the flea evoregions, whereas completeness is the average homogeneity of the flea evoregions with respect to the host evoregions. The *V*-measure, homogeneity, and completeness were calculated using the function ‘vmeasure_calc’ of the R package ‘sabre’ (Nowosad and Stepinski, 2018).

## Results

**Figure 1.**
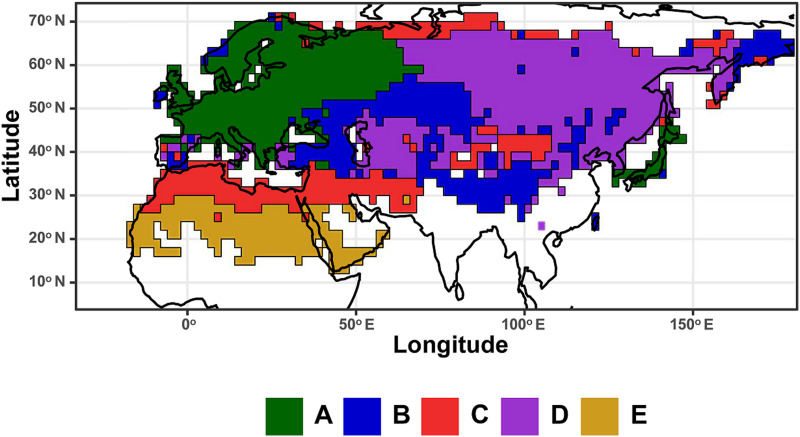
Evoregions (evolutionarily important biogeographic regions) for 206 species of Palearctic fleas. Different evoregions are denoted by different colours.

**Figure 2.**
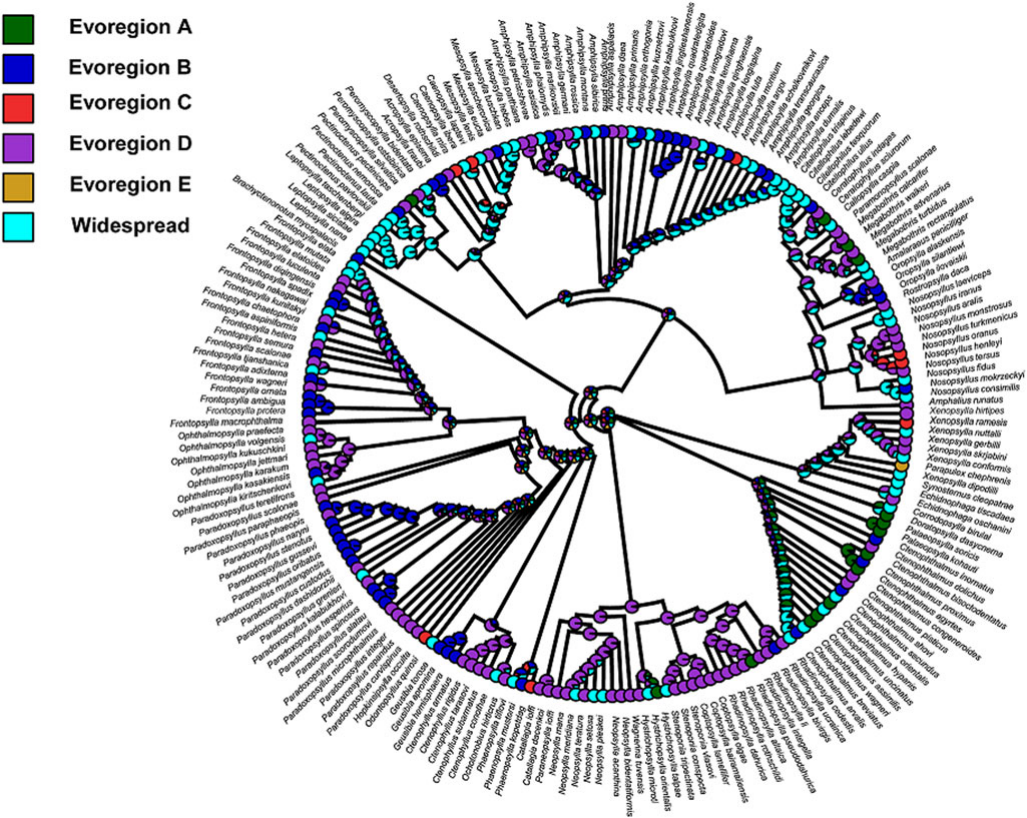
Phylogenetic tree of fleas with colours representing the predominant evoregions (at least 60% of a species' geographic range belongs to a given region). Widespread species are those in which 60% of their geographic range could not be attributed to a single evoregion.

**Figure 3.**
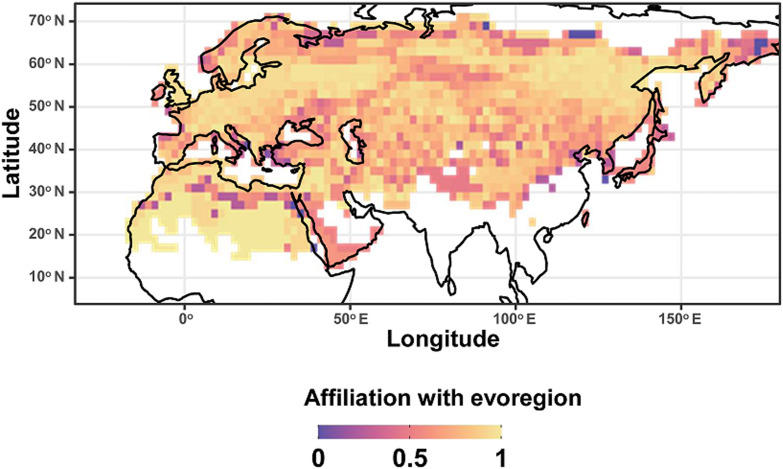
Evolutionary transition zones between flea evoregions.

For fleas, we identified 5 evoregions (Fig. 1). Only one of these evoregions (evoregion E) was spatially continuous. It included the highly arid zones of North Africa and the Arabian Peninsula and was characterized by a single endemic flea (*Parapulex chephrenis*) (Fig. 2), whereas the other fleas inhabiting this evoregion also occurred in other evoregions. Evoregion A covered mainly temperate zones, including (a) almost all of Europe except the southern part of the Iberian Peninsula, the Caucasus, and the south of European Russia; and (b) Japan (Fig. 1). The affiliations of Japan's flea assemblages with this evoregion were weak (Fig. S1; Appendix 5, Supplementary Material). Flea lineages that were diversified in evoregion A were represented mainly by genus *Palaeopsylla* and subgenus *Euctenophthalmus* (Fig. 2). Evoregion B comprised the mountain zones of the Caucasus, western China, and the Chukotka Peninsula, as well northern Kazakhstan (Fig. 1). Evoregion C included the hot deserts of coastal North Africa, the Middle East, part of Mongolia, and the cold deserts of extreme northern Siberia (Fig. 1), although the degree of the affiliations of the Mongolian and northern Siberian flea assemblages was low (Fig. S1; Appendix 5, Supplementary Material). Flea assemblages in both evoregions B and C belonged to a variety of lineages from different families (Fig. 2). Evoregion D included most of Siberia and central Kazakhstan (Fig. 1). Lineages that predominantly diversified in evoregion D were the hystrichopsyllid subfamilies Rhadinopsyllinae and Neopsyllinae, leptopsyllid genera *Ophthalmopsylla* and *Paradoxopsyllus* (albeit only basal taxa of the latter), and fleas of the *Coptopsyllidae* family (Fig. 2). Reconstruction of ancestral states indicated that the majority of lineages at the deep phylogenetic level diversified in multiple regions, except some hystrichopsyllids such as *Stenoponia*, *Rhadinopsylla*, and *Catallagia*, as well as *Coptopsyllidae*, which likely diversified in the evoregion to which they belong (Fig. 2). Transition zones (i.e. zones of high phylogenetic turnover) between evoregions identified for fleas were represented by a boundary between (a) the highly arid and the coastal zones of North Africa (i.e. between evoregions B and E), (b) the arctic zones and the temperate zones of Europe and Siberia (i.e. between evoregion C and evoregions A and B, and (c) the Balkan Mountains and the Aegean Sea (i.e. between evoregions D and A to the west and B to the east; Fig. 3).

**Figure 4.**
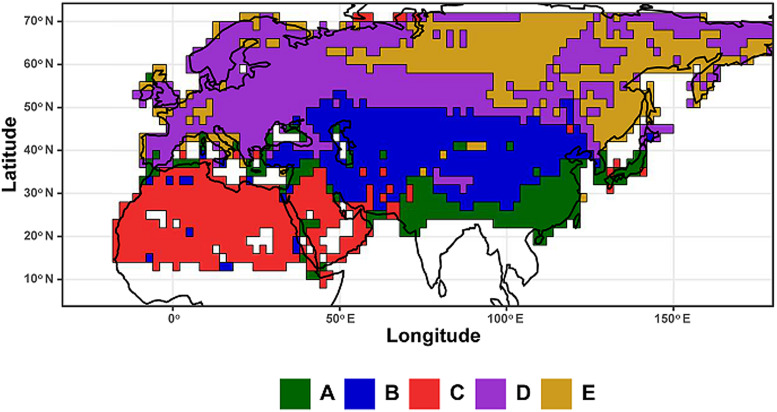
Evoregions (evolutionarily important biogeographic regions) for 265 species of Palearctic small mammals harbouring at least one of 206 flea species for which evoregion regionalization was carried out. Different evoregions are denoted by different colours.

**Figure 5.**
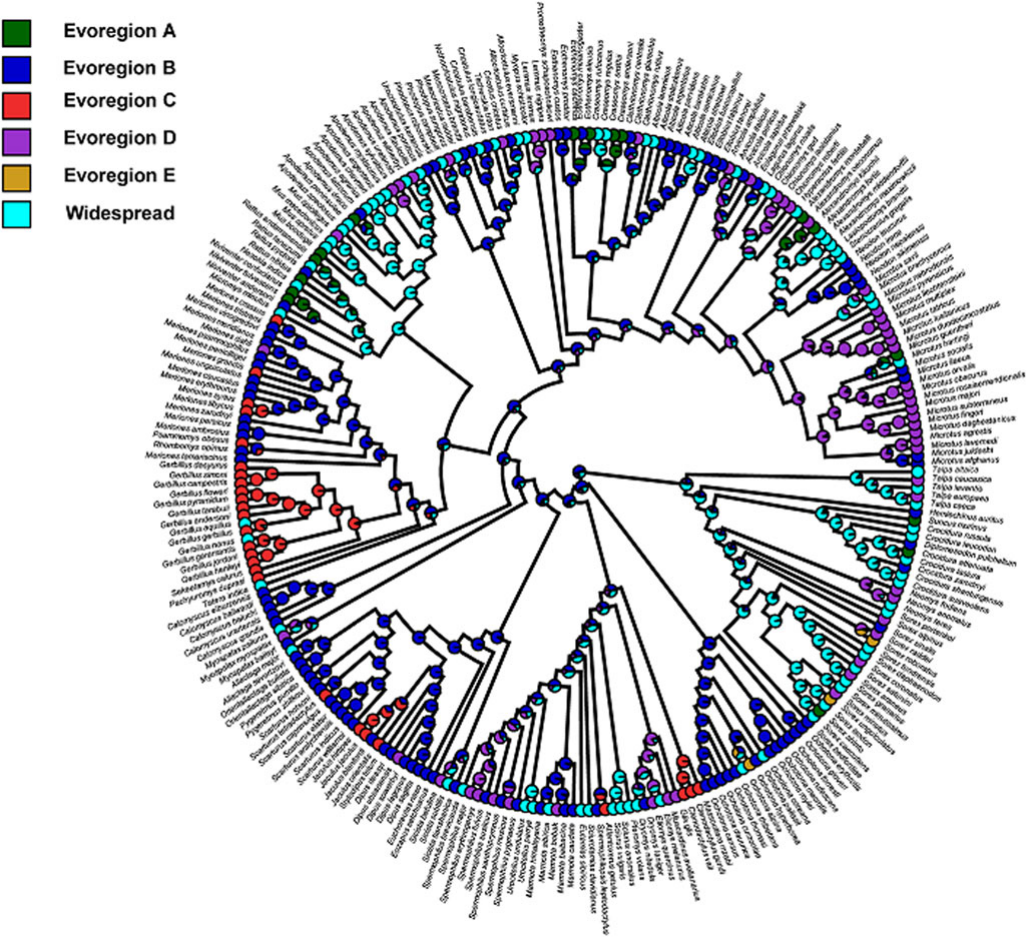
Phylogenetic tree of small mammals harbouring at least one of 206 flea species for which evoregion regionalization was carried out, with colours representing the predominant evoregions (at least 60% of a species' geographic range belongs to a given region). Widespread species are those in which 60% of their geographic range could not be attributed to a single evoregion.

**Figure 6.**
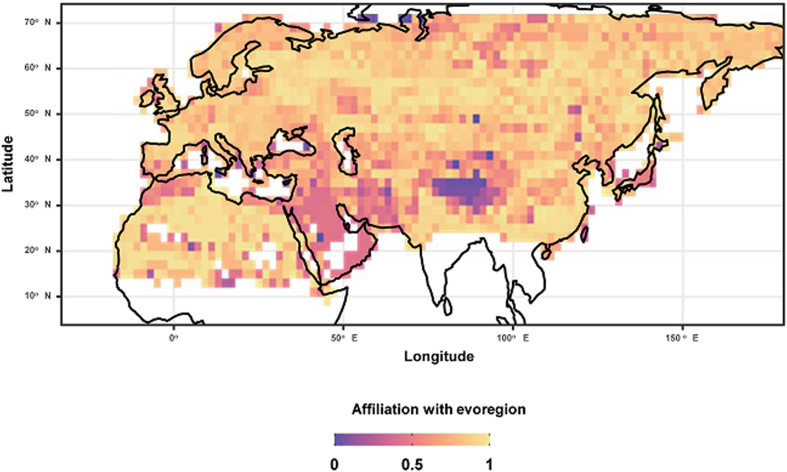
Evolutionary transition zones between small mammal evoregions.

For hosts, 5 evoregions were also identified (Fig. 4). Three of these evoregions (B, C and E) were spatially continuous. Evoregion A mostly covered South Asia including the southern Far East and was associated with the diversification of *Rattus/Niviventer* rats and *Mus* mice, as well as *Eothenomys*, *Craseomys*, and *Alexandromys* arvicolines (Fig. 5). Evoregion B comprised arid and semi-arid Central Asia (including Anatolia, Turan, Iran, Kazakhstan, Mongolia, and Northwestern China). Lineages that diversified in this evoregion were mainly represented by true and mouse-like hamsters (Cricetinae and Calomyscidae, respectively), *Alticola, Ellobius*, and *Neodon* voles, the majority of jerboas (Dipodidae; except *Jaculus*), pikas (Ochotonidae), and gerbils of the Rhombomyina subtribe (Fig. 5). Evoregion C included North Africa and the Arabian Peninsula and was characterized as the diversification centre for *Gerbillus* gerbils, gundis (Ctenodactylidae), and *Jaculus* jerboas (Fig. 5). Evoregion D comprised Europe, southern and northernmost Siberia, the northern Far East, and part of eastern Siberia (except its easternmost portion). Lineages that diversified in evoregion D were lemmings (*Lemmini*), *Chionomys* and *Microtus* voles, dormice (Gliridae), some ground squirrels (*Spermophilus*), and water shrews (*Neomys*; Fig. 5). Finally, evoregion E comprised central Siberia and its easternmost part. It was difficult to discern the clades that radiated there; although this seemed to be the case for *Sorex* shrews, many of these shrews were identified as widespread (Fig. 5). Reconstruction of the ancestral states suggested that radiation of the clades classified to evoregions B, C and D took place in these evoregions (respectively), whereas species belonging to the remaining evoregions radiated elsewhere, and their communities in evoregions A and E were assembled *via* dispersal. This is partly supported by the low affiliation values of many assemblages of evoregion E (Fig. S2; Appendix 5, Supplementary Material), suggesting high phylogenetic heterogeneity within this evoregion. Transition zones between evoregions identified for small mammals are presented in Fig. 6. The transition zone between evoregions B and C covers the deserts of the Arabian Peninsula and the Zagros Mountains. The transition zone between evoregions A and B is represented by the Himalayas, whereas the transition zone between evoregions B and D occurs at the Atlas Mountains and the Strait of Gibraltar. In contrast, no transition zone can be envisaged between evoregions D and E.

The *V*-measure (Nowosad and Stepinski, 2018) was 0.33, with homogeneity being 0.33 and completeness being 0.34. This indicated relatively low similarity between regionalizations for fleas and hosts.

## Discussion

We found that evoregions for fleas and their small mammalian hosts, in general, did not match. In other words, evolutionarily important bioregions for fleas and hosts did not coincide. Obviously, host diversification did not depend on flea diversification. However, the opposite is definitely true (Traub, 1980; Whiting et al., 2008). This, however, does not mean that flea diversification strictly follows that of hosts because, otherwise, the phylogenies of fleas and hosts, across all of the trees, would be highly congruent, whereas this is generally not the case (Krasnov and Shenbrot, 2002; Lu and Wu, 2005; Llaberia-Robledillo et al., 2023). Moreover, Llaberia-Robledillo et al. (2023) analysed cophylogenetic congruence and incongruence between 130 small mammalian species and 202 flea species and found that the degree of cophylogenetic congruence was concentrated in some (e.g. the majority of rodents), but not other (e.g. pikas and shrews), host clades, whereas the degree of phylogenetic congruence and incongruence of fleas greatly varied within clades (i.e. families, subfamilies, and genera). This indicated that codiversification with hosts was characteristic for some, but not other, flea lineages even if they belong to the same higher taxon (e.g. family). As a result, some flea lineages exploit an extremely narrow range of closely related hosts, whereas other confamilial flea lineages exploit hosts of various phylogenetic positions (Traub, 1985). For example, the ceratophyllid genus *Oropsylla* exclusively parasitizes marmotine rodents, whereas hosts of the ceratophyllid *Thrassis* include a variety of rodent families: Sciuridae, Heteromyidae and Muridae. Exploitation of hosts belonging to different phylogenetic lineages by fleas belonging to the same lineage suggests that host switching was one of the most frequent events in flea evolutionary history. In the framework of this study, frequent host switching seemed to be the main reason explaining weak (if present at all) matching between flea and host evoregions.

The main mechanism of host switching is the so-called ecological fitting (Brooks et al., 2006; Hoberg and Brooks, 2008; Agosta et al., 2010; Araujo et al., 2015). Ecological fitting (Janzen, 1985) presents a scenario in which a species interacts with its environment in a way resembling a shared evolutionary history, whereas in reality, the traits allowing the species to exploit this environment evolved in a different place and as a response to different conditions. For parasites, ecological fitting may occur if its main requirement is the resource *sensu stricto* rather than its natural carrier (a given host species). Consequently, if this resource is shared by many host species, a parasite tracking the resource may (1) invade new areas where the resource is present, but where its original host is not; (2) switch to a co-occurring but novel host species if the original host is extinct or develops novel defence tools (Nuismer and Thompson, 2006); and (3) switch to an invading host (Brooks et al., 2006). Obviously, the main resources required by fleas from their hosts are food (i.e. hosts' blood) and a place for pre-imaginal stages to develop (burrow/nest/den). Although these resources are shared by many small mammals, fleas' patterns of acquiring these resources often differ, even between closely related hosts. For example, a flea's acquisition of a host's blood depends not only on the host's morphological traits (e.g. skin thickness), which are similar in close relatives (e.g. Sokolov, 1982), but also on the host's defensive abilities (anti-parasitic grooming or immune response), which may differ between close relatives (Khokhlova et al., 2004; Goüy de Bellocq et al., 2006). A flea's utilization of a host's shelter as a place for pre-imaginal development depends on the shelter's microclimate (because fleas are sensitive to temperature and relative humidity; Marshall, 1981; Krasnov, 2008), which is determined by shelter architecture, as well as the soil and vegetation structure of the host's habitat (Krasnov et al., 1998). Therefore, the probability of host switching is driven not only by the co-occurrence of a potential host and a given parasite but also by the suitability of this host's traits for this parasite, which, in turn, depends on the traits of the parasite itself. Indeed, Krasnov *et al*. (2016) showed that fleas possessing a certain combination of traits, independent of their phylogenetic affinities, utilized hosts that were also characterized by certain trait combinations, although fleas exploiting the same host species were more phylogenetically related than expected by chance. In other words, the species compositions of fleas' host spectra, as well as the species composition of hosts' flea assemblages, are driven by complex interactions between the phylogenies and the traits of both fleas and hosts.

Affiliations of flea species with evoregions and our attempts to reconstruct ancestral geographic distributions (Fig. 2) indicated mostly ex-situ flea diversification, even at the family level. Assuming the monophyly of a majority of flea families (except Hystrichopsyllidae; Zhu et al., 2015), this suggested that (a) initial diversification of these families took place out of the Palearctic and that (b) dispersal was the most likely assembly mechanism of flea communities in evoregions. In fact, the Gondwanan (Australia and South America, terrestrially connected *via* Antarctica until the upper Eocene) origin of the majority of flea families is now commonly accepted (Medvedev, 1996, 2005; Zhu et al., 2015). However, many flea clades rapidly radiated only after their ancestors migrated to Eurasia and Africa (Zhu *et al*., 2015). On the one hand, this suggests that if evoregions for fleas were identified at larger (e.g. global) scales, the geographic distribution of evoregions would differ from that of the smaller scale (e.g. the Palearctic as in this study). Indeed, the distribution of the evoregions identified for the majority of muroid rodents by Maestri and Duarte (2020) at a global scale substantially differed from those of the small mammalian hosts in our study (although muroids constitute the major proportion of them) and, to some extent, matched biogeographic realms. Moreover, the reconstruction of ancestral evoregions for muroids demonstrated that *ex situ* diversification was characteristic of many lineages at the deep phylogenetic level. On the other hand, consideration of evoregions at a smaller scale allows a better and more detailed understanding of geographic patterns of diversification for a given clade at the shallow phylogenetic level. Identification of evoregions for fleas at a global scale warrants further investigation. This will require a gargantuan effort to model the geographic ranges of the majority of flea species (more than 2500; Medvedev, 2005) and to construct their molecular phylogeny. However, the data needed for this endeavour are largely unavailable.

No indication of in-situ diversification supports the results of Gibert et al. (2021), who applied a permutation-based algorithm to infer the relative roles of niche-based *vs* dispersal-based mechanisms in the assembly of regional flea communities in 4 biogeographic realms. They found that these communities' assembly was, to a great extent, governed by dispersal processes and, to a much lesser extent, by niche-based processes. The role of dispersal processes in the assembly of flea and host communities in evoregions is supported, albeit indirectly, by the occurrence of evolutionary transition zones. These zones were most likely determined by natural boundaries, such as deserts or mountain ranges, that could act as barriers to dispersal. This seems to be the case for hosts but not necessarily for fleas, because some of the transition zones detected for them did not correspond to landscape boundaries. For example, an occurrence of a transition zone between evoregions E and C in North Africa probably represented a transition between the climates of the Mediterranean coast (more humid) and the Sahara Desert (highly arid), so that flea species with pre-imagoes highly sensitive to desiccation inhabited evoregion C but not E. Similarly, the transition zone of extreme northern Eurasia could be associated with the sharp difference in soil temperature between evoregions C and A/D. The phylogenetic conservatism of either the degree of sensitivity to environmental factors or the preferable temperature/humidity regime in fleas has never been studied, but similar seasonality of reproduction and activity in many closely related fleas (Darskaya, 1970; Krasnov, 2008) suggests that this may be the case.

In conclusion, a comparison of biogeographic regionalization, coupled with phylogenetic information, between parasites and their hosts gives insight into the shared patterns and processes of 2 different histories. In particular, our study demonstrated that the application of the evoregion approach allowed a better understanding of the contribution of dispersal to cophylogenetic patterns.

## Supporting information

Krasnov and Shenbrot supplementary material Krasnov and Shenbrot supplementary material

## Data Availability

Raw data can be obtained from the corresponding author upon request.

## Supplementary material

The supplementary material for this article can be found at https://doi.org/10.1017/S0031182023000884
