# Supplementary material for: Evoregions of fleas and their small mammalian hosts: Do they coincide?
Source: Parasitology. 2023 Sep 14;150(11):1031–9. doi: 10.1017/S0031182023000884 (PMC10941218; doi:10.1017/S0031182023000884)

**Appendix 1. List of flea species used in the analyses (in alphabetical order)**

*Acropsylla episema*

*Acropsylla traubi*

*Amalaraeus penicilliger*

*Amphalius runatus*

*Amphipsylla anceps*

*Amphipsylla argoi*

*Amphipsylla asiatica*

*Amphipsylla aspalacis*

*Amphipsylla daea*

*Amphipsylla dumalis*

*Amphipsylla georgica*

*Amphipsylla germani*

*Amphipsylla jingtieshanensis*

*Amphipsylla kalabukhovi*

*Amphipsylla kuznetzovi*

*Amphipsylla longispina*

*Amphipsylla marikovskii*

*Amphipsylla montana*

*Amphipsylla montium*

*Amphipsylla orthogonia*

*Amphipsylla parthiana*

*Amphipsylla petristshevae*

*Amphipsylla phaiomydis*

*Amphipsylla prima*

*Amphipsylla primaris*

*Amphipsylla qinghaensis*

*Amphipsylla quadratedigita*

*Amphipsylla quadratoides*

*Amphipsylla rossica*

*Amphipsylla schelkovnikovi*

*Amphipsylla sibirica*

*Amphipsylla tenuihama*

*Amphipsylla transcaucasica*

*Amphipsylla tuta*

*Amphipsylla vinogradovi*

*Brachyctenonotus myospalacis*

*Caenopsylla ibera*

*Caenopsylla laptevi*

*Caenopsylla mira*

*Callopsylla caspia*

*Catallagia dacenkoi*

*Catallagia ioffi*

*Ceratophyllus indages*

*Ceratophyllus sciurorum*

*Citellophilus lebedewi*

*Citellophilus tesquorum*

*Citellophilus trispinus*

*Citellophilus ullus*

*Coptopsylla bairamaliensis*

*Coptopsylla lamellifer*

*Coptopsylla olgae*

*Corrodopsylla birulai*

*Ctenophthalmus agyrtes*

*Ctenophthalmus arvalis*

*Ctenophthalmus assimilis*

*Ctenophthalmus bisoctodentatus*

*Ctenophthalmus breviatus*

*Ctenophthalmus congeneroides*

*Ctenophthalmus dolichus*

*Ctenophthalmus hypanis*

*Ctenophthalmus inornatus*

*Ctenophthalmus orientalis*

*Ctenophthalmus pisticus*

*Ctenophthalmus proximus*

*Ctenophthalmus secundus*

*Ctenophthalmus shovi*

*Ctenophthalmus uncinatus*

*Ctenophthalmus wagneri*

*Ctenophyllus armatus*

*Ctenophyllus conothae*

*Ctenophyllus rigidus*

*Ctenophyllus subarmatus*

*Ctenophyllus tarasovi*

*Desertopsylla rothschildi*

*Doratopsylla dasycnema*

*Echidnophaga oschanini*

*Echidnophaga tiscadaea*

*Frontopsylla adixterna*

*Frontopsylla ambigua*

*Frontopsylla aspiniformis*

*Frontopsylla chaetophora*

*Frontopsylla diqingensis*

*Frontopsylla elata*

*Frontopsylla elatoides*

*Frontopsylla hetera*

*Frontopsylla kunitskyi*

*Frontopsylla luculenta*

*Frontopsylla macrophthalma*

*Frontopsylla mutata*

*Frontopsylla nakagawai*

*Frontopsylla ornata*

*Frontopsylla protera*

*Frontopsylla scalonae*

*Frontopsylla semura*

*Frontopsylla spadix*

*Frontopsylla tjanshanica*

*Frontopsylla wagneri*

*Geusibia apromina*

*Geusibia hemisphaera*

*Geusibia torosa*

*Hopkinsipsylla occulta*

*Hystrichopsylla microti*

*Hystrichopsylla orientalis*

*Hystrichopsylla talpae*

*Leptopsylla algira*

*Leptopsylla nana*

*Leptopsylla sicistae*

*Leptopsylla taschenbergi*

*Megabothris advenarius*

*Megabothris calcarifer*

*Megabothris rectangulatus*

*Megabothris turbidus*

*Megabothris walkeri*

*Mesopsylla apscheronica*

*Mesopsylla eucta*

*Mesopsylla hebes*

*Mesopsylla lenis*

*Mesopsylla tuschkan*

*Neopsylla acanthina*

*Neopsylla bidentatiformis*

*Neopsylla mana*

*Neopsylla meridiana*

*Neopsylla pleskei*

*Neopsylla setosa*

*Neopsylla teratura*

*Nosopsyllus aralis*

*Nosopsyllus consimilis*

*Nosopsyllus fidus*

*Nosopsyllus henleyi*

*Nosopsyllus iranus*

*Nosopsyllus laeviceps*

*Nosopsyllus mokrzeckyi*

*Nosopsyllus monstrosus*

*Nosopsyllus oranus*

*Nosopsyllus tersus*

*Nosopsyllus turkmenicus*

*Ochotonobius hirticrus*

*Odontopsyllus quirosi*

*Ophthalmopsylla jettmari*

*Ophthalmopsylla karakum*

*Ophthalmopsylla kasakiensis*

*Ophthalmopsylla kiritschenkovi*

*Ophthalmopsylla kukuschkini*

*Ophthalmopsylla praefecta*

*Ophthalmopsylla volgensis*

*Oropsylla alaskensis*

*Oropsylla ilovaiskii*

*Oropsylla silantiewi*

*Palaeopsylla kohauti*

*Palaeopsylla soricis*

*Paradoxopsyllus alatau*

*Paradoxopsyllus curvispinus*

*Paradoxopsyllus custodus*

*Paradoxopsyllus dashidorzhii*

*Paradoxopsyllus grenieri*

*Paradoxopsyllus gussevi*

*Paradoxopsyllus hesperius*

*Paradoxopsyllus integer*

*Paradoxopsyllus kalabukhovi*

*Paradoxopsyllus microphthalmus*

*Paradoxopsyllus mustangensis*

*Paradoxopsyllus naryni*

*Paradoxopsyllus oribatus*

*Paradoxopsyllus paraphaeopis*

*Paradoxopsyllus phaeopis*

*Paradoxopsyllus repandus*

*Paradoxopsyllus scalonae*

*Paradoxopsyllus scorodumovi*

*Paradoxopsyllus spinosus*

*Paradoxopsyllus stenotus*

*Paradoxopsyllus teretifrons*

*Paramonopsyllus scalonae*

*Paraneopsylla ioffi*

*Parapulex chephrenis*

*Pectinoctenus lauta*

*Pectinoctenus nemorosa*

*Pectinoctenus pavlovskii*

*Pectinoctenus pectiniceps*

*Peromyscopsylla bidentata*

*Peromyscopsylla ostsibirica*

*Peromyscopsylla silvatica*

*Phaenopsylla kopetdag*

*Phaenopsylla mustersi*

*Phaenopsylla tiflovi*

*Rhadinopsylla altaica*

*Rhadinopsylla bivirgis*

*Rhadinopsylla cedestis*

*Rhadinopsylla dahurica*

*Rhadinopsylla integella*

*Rhadinopsylla li*

*Rhadinopsylla pseudodahurica*

*Rhadinopsylla rothschildi*

*Rhadinopsylla ucrainica*

*Rostropsylla daca*

*Stenoponia conspecta*

*Stenoponia tripectinata*

*Stenoponia vlasovi*

*Synosternus cleopatrae*

*Wagnerina tuvensis*

*Xenopsylla conformis*

*Xenopsylla dipodilli*

*Xenopsylla gerbilli*

*Xenopsylla hirtipes*

*Xenopsylla nuttalli*

*Xenopsylla ramesis*

*Xenopsylla skrjabini*

**Appendix 2. List of small mammal species used in the analyses (in alphabetical order)**

| *Alexandromys fortis* |
| --- |
| *Alexandromys kikuchii* |
| *Alexandromys maximowiczii* |
| *Alexandromys middendorffii* |
| *Alexandromys montebelli* |
| *Alexandromys oeconomus* |
| *Allactaga major* |
| *Allactaga severtzovi* |
| *Allocricetulus curtatus* |
| *Allocricetulus eversmanni* |
| *Alticola argentatus* |
| *Alticola barakshin* |
| *Alticola lemminus* |
| *Alticola parvidens* |
| *Alticola semicanus* |
| *Alticola stoliczkanus* |
| *Alticola strelzowi* |
| *Apodemus agrarius* |
| *Apodemus argenteus* |
| *Apodemus chevrieri* |
| *Apodemus draco* |
| *Apodemus flavicollis* |
| *Apodemus latronum* |
| *Apodemus mystacinus* |
| *Apodemus peninsulae* |
| *Apodemus ponticus* |
| *Apodemus speciosus* |
| *Apodemus sylvaticus* |
| *Apodemus uralensis* |
| *Apodemus witherbyi* |
| *Arvicola amphibius* |
| *Arvicola italicus* |
| *Arvicola periicus* |
| *Arvicola sapidus* |
| *Atlantoxerus getulus* |
| *Calomyscus bailwardi* |
| *Calomyscus baluchi* |
| *Calomyscus elburzensis* |
| *Calomyscus grandis* |
| *Calomyscus urartensis* |
| *Chionomys gud* |
| *Chionomys lasistanius* |
| *Chionomys nivalis* |
| *Chionomys roberti* |
| *Clethrionomys centralis* |
| *Clethrionomys glareolus* |
| *Clethrionomys rutilus* |
| *Craseomys andersoni* |
| *Craseomys regulus* |
| *Craseomys rufocanus* |
| *Craseomys smithii* |
| *Cricetulus barabensis* |
| *Cricetulus longicaudatus* |
| *Cricetus cricetus* |
| *Crocidura attenuata* |
| *Crocidura lasiura* |
| *Crocidura leucodon* |
| *Crocidura russula* |
| *Crocidura shantungensis* |
| *Crocidura suaveolens* |
| *Crocidura zarudnyi* |
| *Ctenodactylus gundi* |
| *Ctenodactylus vali* |
| *Diplomesodon pulchellum* |
| *Dipus deasyi* |
| *Dipus lagopus* |
| *Dipus sagitta* |
| *Dipus sowerbyi* |
| *Dipus ubsanensis* |
| *Dryomys laniger* |
| *Dryomys nitedula* |
| *Eliomys melanurus* |
| *Eliomys quercinus* |
| *Ellobius fuscocapillus* |
| *Ellobius talpinus* |
| *Ellobius tancrei* |
| *Eolagurus przewalskii* |
| *Eothenomys cachinus* |
| *Eothenomys custos* |
| *Eothenomys eleusis* |
| *Eothenomys melanogaster* |
| *Eothenomys proditor* |
| *Eozapus setchuanus* |
| *Euchoreutes naso* |
| *Eutamias sibiricus* |
| *Gerbillus andersoni* |
| *Gerbillus aquillus* |
| *Gerbillus campestris* |
| *Gerbillus dasyurus* |
| *Gerbillus floweri* |
| *Gerbillus garamantis* |
| *Gerbillus gerbillus* |
| *Gerbillus henleyi* |
| *Gerbillus jordani* |
| *Gerbillus nanus* |
| *Gerbillus pyramidum* |
| *Gerbillus simoni* |
| *Gerbillus tarabuli* |
| *Glis glis* |
| *Hemiechinus auritus* |
| *Hyperacrius fertilis* |
| *Jaculus blanfordi* |
| *Jaculus hirtipes* |
| *Jaculus jaculus* |
| *Jaculus orientalis* |
| *Lagurus lagurus* |
| *Lasiopodomys brandtii* |
| *Lemmus lemmus* |
| *Lemmus nigripes* |
| *Marmota baibacina* |
| *Marmota bobak* |
| *Marmota caudata* |
| *Marmota himalayana* |
| *Marmota sibirica* |
| *Massoutiera mzabi* |
| *Meriones ambrosius* |
| *Meriones caucasius* |
| *Meriones crassus* |
| *Meriones dahli* |
| *Meriones erythrourus* |
| *Meriones grandis* |
| *Meriones libycus* |
| *Meriones meridianus* |
| *Meriones penicilliger* |
| *Meriones persicus* |
| *Meriones psammophilus* |
| *Meriones syrius* |
| *Meriones tamariscinus* |
| *Meriones tristrami* |
| *Meriones unguiculatus* |
| *Meriones vinogradovi* |
| *Meriones zarudnyi* |
| *Mesocricetus brandti* |
| *Mesocricetus raddei* |
| *Micromys minutus* |
| *Microtus afghanus* |
| *Microtus agrestis* |
| *Microtus arvalis* |
| *Microtus brachycercus* |
| *Microtus daghestanicus* |
| *Microtus duodecimcostatus* |
| *Microtus fingeri* |
| *Microtus guentheri* |
| *Microtus hartingi* |
| *Microtus ilaeus* |
| *Microtus juldashi* |
| *Microtus lavernedi* |
| *Microtus liechtensteini* |
| *Microtus lusitanicus* |
| *Microtus majori* |
| *Microtus multiplex* |
| *Microtus nebrodensis* |
| *Microtus obscurus* |
| *Microtus pyrenaicus* |
| *Microtus rossiaemeridionalis* |
| *Microtus savii* |
| *Microtus socialis* |
| *Microtus subterraneus* |
| *Microtus tatricus* |
| *Mus booduga* |
| *Mus macedonicus* |
| *Mus spicilegus* |
| *Mus spretus* |
| *Muscardinus avellanarius* |
| *Myopus schisticolor* |
| *Myospalax baileyi* |
| *Myospalax myospalax* |
| *Myospalax psilurus* |
| *Neodon irene* |
| *Neodon leucurus* |
| *Neodon nepalensis* |
| *Neodon sikimensis* |
| *Neomys anomalus* |
| *Neomys fodiens* |
| *Neomys teres* |
| *Nesokia indica* |
| *Niviventer andersoni* |
| *Niviventer confucianus* |
| *Niviventer fulvescens* |
| *Nothocricetulus migratorius* |
| *Ochotona alpina* |
| *Ochotona cansus* |
| *Ochotona coreana* |
| *Ochotona curzoniae* |
| *Ochotona dauurica* |
| *Ochotona erythrotis* |
| *Ochotona forresti* |
| *Ochotona gloveri* |
| *Ochotona hyperborea* |
| *Ochotona macrotis* |
| *Ochotona pallasii* |
| *Ochotona roylei* |
| *Ochotona rufescens* |
| *Ochotona thibetana* |
| *Ochotona thomasi* |
| *Orientallactaga bullata* |
| *Orientallactaga sibirica* |
| *Pachyuromys duprasi* |
| *Phodopus campbelli* |
| *Phodopus roborovskii* |
| *Phodopus sungorus* |
| *Prometheomys schaposchnikowi* |
| *Psammomys obesus* |
| *Pteromys volans* |
| *Pygeretmus pumilio* |
| *Pygeretmus zhitkovi* |
| *Rattus andamanensis* |
| *Rattus nitidus* |
| *Rattus pyctoris* |
| *Rattus tanezumi* |
| *Rhombomys opimus* |
| *Scarturus aralychensis* |
| *Scarturus caprimulgus* |
| *Scarturus elater* |
| *Scarturus hotsoni* |
| *Scarturus indicus* |
| *Scarturus tetradactylus* |
| *Scarturus williamsi* |
| *Sciurotamias davidianus* |
| *Sciurus anomalus* |
| *Sciurus vulgaris* |
| *Sekeetamys calurus* |
| *Sicista betulina* |
| *Sicista subtilis* |
| *Sicista tianshanica* |
| *Sorex alpinus* |
| *Sorex araneus* |
| *Sorex bedfordiae* |
| *Sorex caecutiens* |
| *Sorex coronatus* |
| *Sorex daphaenodon* |
| *Sorex granarius* |
| *Sorex isodon* |
| *Sorex minutissimus* |
| *Sorex minutus* |
| *Sorex portenkoi* |
| *Sorex raddei* |
| *Sorex roboratus* |
| *Sorex satunini* |
| *Sorex shinto* |
| *Sorex sinalis* |
| *Sorex tundrensis* |
| *Sorex unguiculatus* |
| *Spermophilopsis leptodactylus* |
| *Spermophilus brevicauda* |
| *Spermophilus erythrogenys* |
| *Spermophilus fulvus* |
| *Spermophilus major* |
| *Spermophilus musicus* |
| *Spermophilus pygmaeus* |
| *Spermophilus suslicus* |
| *Spermophilus xanthoprymnus* |
| *Stenocranius gregalis* |
| *Stylodipus telum* |
| *Suncus murinus* |
| *Talpa altaica* |
| *Talpa caeca* |
| *Talpa caucasica* |
| *Talpa europaea* |
| *Talpa levantis* |
| *Tatera indica* |
| *Tscherskia triton* |
| *Urocitellus parryii* |
| *Urocitellus undulatus* |
| *Urocricetulus kamensis* |

**Appendix 3. Methodology of geographic range estimation**

Records of occurrences of fleas and rodents for Species Distribution Modelling (SDM) were taken from the Global Biodiversity Information Facility (GBIF: https://www.gbif.org), Finnish Biodiversity Information Facility (https://laji.fi), databases of the following collections, with some available online and others not. Collections available online were (a) the mammalogical collection of the Department of Biogeography, Faculty of Geography, Moscow State University (<https://www.biogeo.ru/index.php/elektronnyj-katalog/katalog-zoologicheskoj-kollektsii-mlekopitayushchie>), (b) the Museum of the Archaeology, Ethnography and Ecology of Siberia, Kemerovo State University (<https://museum.kemsu.ru/catalog>), and (c) the Siberian Zoological Museum of the Institute of Animal Systematics and Ecology, Siberian Branch of the Russian Academy of Sciences (Novosibirsk, Russia) (<https://szmn.eco.nsc.ru/Vertebr/Mammalia.htm>). We also used data from the collections of the Naturhistorisches Museum, Wien (provided by F. Zachos, K. Stefke and A. Bibl), the Senckenberg Naturmuseum, Frankfurt (provided by K. Krohmann), the Slovenian Museum of Natural History, Ljubljana (provided by B. Kryštufek), the Natural History Museum, Prague (provided by B. Kryštufek), and the Zoological Museum of Moscow State University (provided by V. Lebedev), as well as from various scientific publications. Most of these data had no original GPS coordinates and were geo-referenced using Geographic Names Gazetteers available at http://earth-info.nga.mil/gns/html/cntry_files.html and checked for suitable habitats using Google Earth. Data that could not be precisely geo-referenced (± 5 km) were excluded from the analysis.

Environmental data for SDM were used as 30 arc-second grids (approximately 1 km resolution) across the entire Palearctic. These data included climate, relief, and vegetation variables. The climate variables (annual mean temperature, mean daily temperature range, maximal temperature of warmest month, minimal temperature of coldest month, temperature annual range, and precipitation of winter, spring, summer, and autumn months) were obtained from WORLDCLIM Version 2.0 (Fick and Hijmans, 2017), available at http://www.worldclim.org /version2. Altitudes were extracted from the GOTOPO30 data set available at https://lta.cr.usgs.gov/GTOPO30. Slope data were derived from altitude using the Spatial Analyst module of ArcGIS 10.8.2. The data on abundance of green vegetation (NDVI index) were obtained from the VEGETATION Programme (http://free.vgt.vito.be; data for 1998-2007, each 10-day estimations) and averaged for winter, spring, summer, and autumn months.

The SDMs were built with MAXENT 3.4.0 software (Phillips *et al*., 2006). The extent of the study area or the “landscape of interest” significantly affects the SDM results (Anderson and Raza, 2010; Elith *et al*., 2011). To define the study area for a given species, we calculated the kernel density of this species’ occurrence points with a search radius equal to 4°, reclassified the obtained raster so that the original kernel density values equal to or more than 0.05 were converted to 1 and values less than 0.05 to "NoData", and used this reclassified raster as the mask for clipping environmental variables to the study area. Models were constructed with default MAXENT settings as these settings were demonstrated to be the most appropriate for wide-ranging data (Phillips and Dudik, 2008; Warren and Seifert ,2011). We used the MAXENT logistic output, which provides estimates of relative habitat suitability (Elith *et al*., 2011).

To delineate the areas of real species occurrence, the original model values, ranging continuously from 0 to 1, were transformed to a binary 0 or 1 using a threshold value. The threshold value was chosen to be equal to the “maximum training sensitivity plus specificity”; it was demonstrated experimentally (Liu *et al*., 2013) that this threshold provides optimal results. After reclassifying the original raster according to the chosen threshold value, the reclassified raster was transformed into polygons. Only polygons containing occurrence records were considered as areas of occurrence. Polygons of geographic ranges were transformed to grids with resolution 0.5 × 0.5 decimal degrees for widely distributed species and to grids with resolution 0.25 × 0.25 decimal degrees for narrowly distributed species, and these grids were then resampled to resolution 2 × 2 decimal degrees. All map operations were performed using ArcGIS 10.8.2 software.

References

**Anderson RP, Raza A** (2010) The effect of the extent of the study region on GIS models of species geographic distributions and estimates of niche evolution: preliminary tests with montane rodents (genus *Nephelomys*) in Venezuela. *Journal of Biogeography* **37**, 1378-1393.

**Elith J, Phillips SJ, Hastie T, Dudík M, Chee YE, Yates CJ** (2011) A statistical explanation of MaxEnt for ecologists. *Diversity and Distributions* **17**, 43–57.

**Fick SE, Hijmans RJ** (2017) WorldClim 2: new 1-km spatial resolution climate surfaces for global land areas. *International Journal of Climatology* **37**, 4302–4315.

**Liu C, White M, Newell G** (2013) Selecting thresholds for the prediction of species occurrence with presence-only data. *Journal of Biogeography* **40**, 778–789.

**Phillips SJ, Dudík M** (2008) Modeling of species distributions with Maxent: new extensions and a comprehensive evaluation. *Ecography* **31**, 161–175.

**Phillips SJ, Anderson RP, Schapire RE** (2006) Maximum entropy modeling of species geographic distributions. *Ecological Modelling* **190**, 231-259.

**Warren DL, Seifert SN** (2011) Ecological niche modeling in Maxent: the importance of model complexity and the performance of model selection criteria. *Ecological Applications* **21**, 335–342.

**Appendix 4. Sources of information on the phylogenetic positions of rodent species absent from the phylogenetic tree of Upham *et al.* (2019)**

Topological positions of rodent species absent from the phylogenetic tree of Upham et al. (2019) were taken from Lebedev *et al*. (2022) for Allactaginae; Lebedev *et al*. (2018) and Lisenkova *et al.* (2023) for the *Dipus sagitta* species complex; Shenbrot *et al.* (2016) for the *Jaculus jaculus* species complex; Kryštufek and Shenbrot (2022) for Arvicolinae; Bouarakia *et al.* (2018) for the *Gerbillus henleyi* species complex; Pavlinov *et al.* (2010) and Dianat *et al*. (2020) for the *Meriones libycus* species complex; Ito et al. (2010), Nanova (2014) and Nanova *et al.* (2020) for the *Meriones meridianus* species complex; and Dianat *et al.* (2016) for the *Meriones persicus* species complex.

References

**Bouarakia O, Denys C, Nicolas V, Tifarouine L, Benazzou T, Benhoussa A** (2018) Notes on the distribution and phylogeography of two rare small Gerbillinae (Rodentia, Muridae) in Morocco: *Gerbillus simoni* and *Gerbillus henleyi*. *Comptes Rendus Biologies* **341**, 398-409.

**Dianat M, Darvish J, Aliabadian M, Haddadian H, Khajeh A, Nicolas V** (2016) Integrative taxonomy of *Meriones persicus* (Rodentia, Gerbillinae) in Iran. *Iranian Journal of Animal Biosystematics* **12**, 77-95.

**Dianat M, Darvish J, Aliabadian M, Siahsarvie R, Krystufek B, Nicolas V** (2020) Systematics and evolution of the libyan jird based on molecular and morphometric data. Journal of Zoological Systematics and Evolutionary Research 58, 439-458.

**Ito M, Jiang W, Sato JJ, Zhen Q, Jiao W, Goto K, Sato H, Ishiwata K, Oku Y, Chai J-J, Kamiya H** (2010) Molecular phylogeny of the subfamily Gerbillinae (Muridae, Rodentia) with emphasis on species living in the Xinjiang-Uygur Autonomous Region of China and based on the mitochondrial cytochrome *b* and cytochrome *c* oxidase subunit ii genes. *Zoological Science* **27**, 269–278.

**Kryštufek B, Shenbrot G** (2022) *Voles and Lemmings (Arvicolinae) of the Palaearctic Region*. Maribor, Slovenia: University of Maribor, University Press.

**Lebedev VS, Shenbrot GI, Krystufek B, Mahmoudi A, Melnikova MN, Solovyeva EN, Lisenkova AA, Undrakhbayar E, Rogovin KA, Surov AV, Bannikova AA** (2022) Phylogenetic relations and range history of jerboas of the Allactaginae subfamily (Dipodidae, Rodentia). *Scientific Reports* **12**, 842-857.

**Lebedev VS, Bannikova AA, Lu L, Snytnikov EA, Yansanjav A, Solovyeva EA, Abramov AV, Surov AV, Shenbrot GI** (2018) Phylogeographical study reveals high genetic diversity in a widespread desert rodent, *Dipus sagitta* (Dipodidae: Rodentia). *Biological Journal of the Linnean Society* **123**, 445-462.

**Lisenkova AA, Lebedev VS, Undrakhbayar E, Bogatyreva VY, Melnikova MN, Nazarov RA, Rogovin KA, Surov AV, Shenbrot GI, Bannikova AA** (2023) Phylogeny of the *Dipus sagitta* species complex by nuclear gene sequences. *Doklady Biological Sciences* **509**, 135–139.

**Nanova OG** (2014) Geographical variation in the cranial measurements of the midday jird *Meriones meridianus* (Rodentia: Muridae) and its taxonomic implications. *Journal of Zoological Systematics and Evolutionary Research* **52**, 75-85.

**Nanova OG, Lebedev VS, Matrosova VA, Adiya Y, Undrakhbayar E, Surov AV, Shenbrot GI** (2020) Phylogeography, phylogeny, and taxonomical revision of the Midday jird (*Meriones meridianus*) species complex from Dzungaria*. Journal of Zoological Systematics and Evolutionary Research* **58**, 1335-1358.

**Pavlinov IY, Lissovskiy AA, Obolenskaya EV** (2010) Geographic variation of skull traits in the Libyan jird, *Meriones libycus* (Rodentia: Gerbillinae), over its entire distribution area. *Russian Journal of Theriology* **9**, 19-26 (in Russian).

**Shenbrot GI, Feldstein I, Meiri S** (2016) Are cryptic species of the Lesser Egyptian Jerboa, *Jaculus jaculus* (Rodentia, Dipodidae), really cryptic? Re-evaluation of their taxonomic status with new data from Israel and Sinai. *Journal of Zoological Systematics and Evolutionary Research* **54**, 148-159.

**Appendix 5. Supplementary figures**

**Fig. S1.** Evoregions for 206 species of Palearctic fleas and the degree of each flea assemblage’s affiliation, in each 2° × 2° grid cell, with a respective evoregion (a weaker affiliation is indicated by a lighter colour).


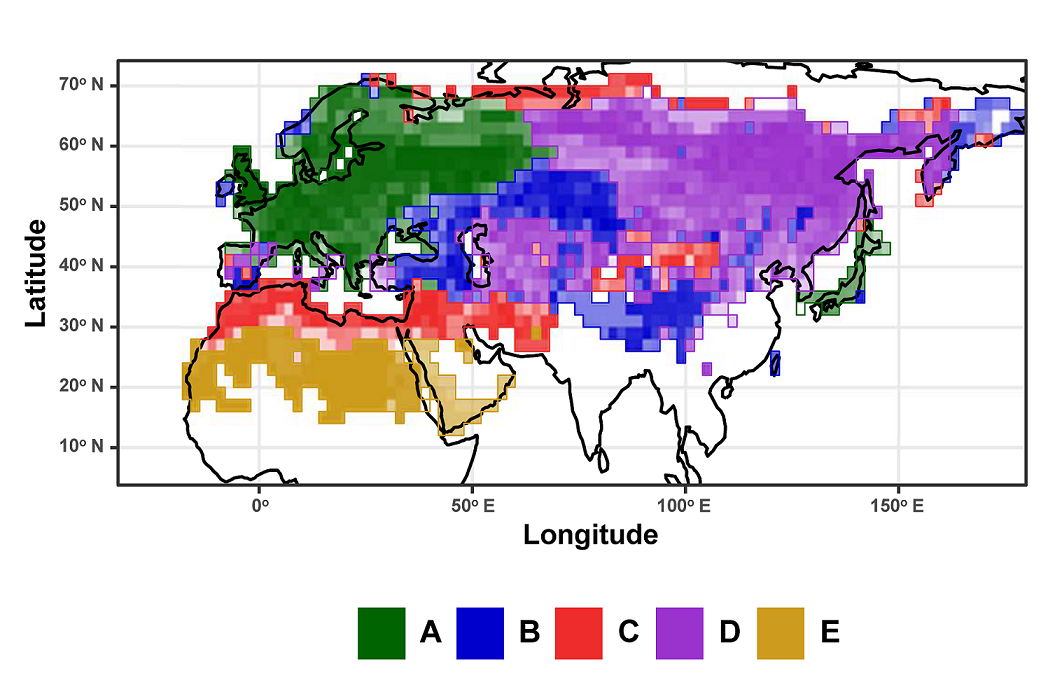


**Fig. S2.** Evoregions for 265 species of Palearctic small mammals and each assemblage’s degree of affiliation, in each 2° × 2° grid cell, with a respective evoregion (a weaker affiliation is indicated by a lighter colour).


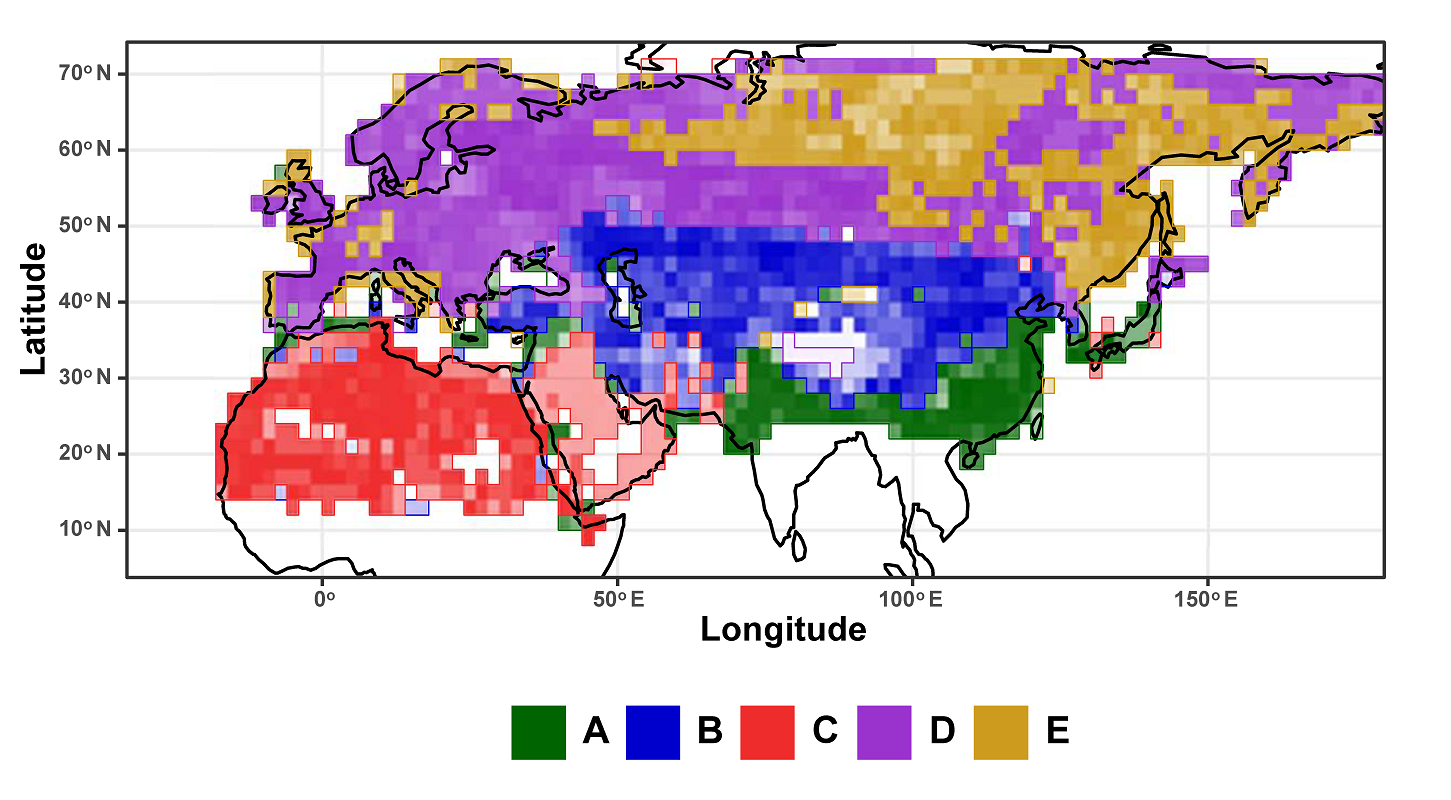

Supplement: Krasnov and Shenbrot supplementary material [file S0031182023000884sup001.docx]
